# Targeting mixed lineage kinases in ER-positive breast cancer cells leads to G2/M cell cycle arrest and apoptosis

**DOI:** 10.18632/oncotarget.1093

**Published:** 2013-07-07

**Authors:** Limin Wang, Kathleen A. Gallo, Susan E. Conrad

**Affiliations:** ^1^ Department of Microbiology and Molecular Genetics, Michigan State University, East Lansing MI; ^2^ Department of Physiology, Michigan State University, East Lansing MI

**Keywords:** breast cancer, kinase inhibitors, cell cycle, apoptosis

## Abstract

Estrogen receptor (ER)-positive tumors represent the most common type of breast cancer, and ER-targeted therapies such as antiestrogens and aromatase inhibitors have therefore been widely used in breast cancer treatment. While many patients have benefited from these therapies, both innate and acquired resistance continue to be causes of treatment failure. Novel targeted therapeutics that could be used alone or in combination with endocrine agents to treat resistant tumors or to prevent their development are therefore needed. In this report, we examined the effects of inhibiting mixed-lineage kinase (MLK) activity on ER-positive breast cancer cells and non-tumorigenic mammary epithelial cells. Inhibition of MLK activity with the pan-MLK inhibitor CEP-1347 blocked cell cycle progression in G2 and early M phase, and induced apoptosis in three ER-positive breast cancer cell lines, including one with acquired antiestrogen resistance. In contrast, it had no effect on the cell cycle or apoptosis in two non-tumorigenic mammary epithelial cell lines. CEP-1347 treatment did not decrease the level of active ERK or p38 in any of the cell lines tested. However, it resulted in decreased JNK and NF-κB activity in the breast cancer cell lines. A JNK inhibitor mimicked the effects of CEP-1347 in breast cancer cells, and overexpression of c-Jun rescued CEP-1347-induced Bax expression. These results indicate that proliferation and survival of ER-positive breast cancer cells are highly dependent on MLK activity, and suggest that MLK inhibitors may have therapeutic efficacy for ER-positive breast tumors, including ones that are resistant to current endocrine therapies.

## INTRODUCTION

Breast cancer is the most frequent cancer type and the second most common cause of cancer death in women in the US [1]. Approximately 70% of breast tumors are estrogen receptor alpha (ER)-positive and these tumors usually depend on estrogen for growth [2]; thus, ER-targeted endocrine therapies are useful for the treatment of patients with ER^+^ breast tumors [3]. Although many patients benefit from endocrine therapy, intrinsic or acquired resistance remains a serious problem. Multiple mechanisms underlying endocrine resistance have been proposed, including deregulation of ER activity, altered cell cycle and cell survival signaling, and aberrant activation of growth factor pathways [4]. Among these, activation of growth factor signaling is the best characterized and strategies to circumvent endocrine resistance have focused on co-targeting growth factor pathways and ER. For example, drugs targeting HER2, IGF-1R and PI3K/Akt/mTOR have been tested in clinical trials as monotherapies or in combination with endocrine agents for ER^+^ tumors [5-7]. Blocking a single receptor or pathway may only be effective for a subset of tumors, however, and targeting key signaling nodes common to multiple receptors and pathways may provide therapeutic advantages.

Mitogen-activated-protein-kinase (MAPK) pathways transduce a large variety of external signals, leading to cellular responses including proliferation, differentiation, inflammation and apoptosis [8, 9]. Several studies have demonstrated cross talk between MAPK pathways and ER. For example, ER activates MAPK pathways via nongenomic mechanisms; and MAPKs can mediate posttranslational modifications of ER and its coregulators, influencing both ER activity and sensitivity of ER^+^ breast cancer cells to endocrine therapies [10]. In addition, increased ERK1/2 activity or p38 kinase activity is reportedly associated with endocrine resistance [11-13]. Thus, MAPK pathway components may be useful molecular targets for overcoming or preventing endocrine resistance in ER^+^ breast cancer.

The mixed-lineage kinases (MLKs) are a family of serine/threonine kinases that relay signals from cell surface receptors to MAPKs. The MLK family is comprised of three subgroups: MLKs (MLK1-4), the dual leucine zipper-bearing kinase(s) (DLKs), and zipper sterile-α-motif kinase (ZAK) [14]. MLKs can function as MAP Kinase Kinase Kinases (MAP3Ks) to phosphorylate and activate Map Kinase Kinases (MAP2Ks), which in turn phosphorylate and activate MAPKs, including c-Jun N-terminal kinases (JNKs), p38, and extracellular signal-regulated kinases (ERKs). Active MAPKs phosphorylate multiple nuclear and cytosolic substrates. Within the MLK subfamily, the best characterized is MLK3, which can activate JNK through MAP2K4/7, and p38 through MAP2K3/6. In addition, MLK3 is proposed to act as a critical scaffold for activation of the MAP3K B-Raf, leading to ERK activation. Thus, MLK3-mediated ERK activation does not depend upon MLK3 kinase activity [15].

Consistent with their ability to activate multiple MAPK pathways, MLKs mediate diverse biological processes. Early studies reported that MLK3 promotes neuronal apoptosis, and that apoptosis triggered by MLK3 overexpression in neurons is mediated through JNK [16]. In contrast, MLK3 promotes proliferation and survival in other cell types. Overexpression of MLK3 induces transformation and anchorage-independent growth of NIH 3T3 fibroblasts [17]. RNA interference experiments indicate that MLK3 is required for proliferation/survival of various cancer cell lines including colon, ovarian [15] and breast cells [18], and for migration/invasion of ovarian [19], triple negative/basal breast [18, 20, 21] and gastric carcinoma cells [22].

The downstream targets through which MLKs mediate their effects are not completely characterized. One major downstream target of MLK3 is JNK, which, upon activation, phosphorylates nuclear substrates including c-Jun. c-Jun, a major component of the activator protein 1 (AP-1) transcription factor, regulates the expression of many genes involved in cell proliferation and survival [23]. MLK3 can also activate nuclear factor kappa B (NF-κB) through phosphorylation of IκB kinase alpha (IKKα) and IKKβ [24].

The fact that MLKs promote processes such as proliferation, survival and migration suggests that they might be viable targets for cancer therapeutics. CEP-1347 and CEP-11004 are ATP analogues that selectively inhibit the MLK family of kinases [25, 26]. In this study, we investigated the effects and mechanisms of action of CEP-1347 in ER^+^ breast cancer cells and non-tumorigenic mammary epithelial cells. Our data demonstrate that CEP-1347 induces G2/M arrest, failure in metaphase entry, and apoptosis in three different ER^+^ breast cancer cell lines, including one with acquired endocrine resistance. In contrast, CEP-1347 has minor effects on two non-tumorigenic mammary epithelial cell lines. Mechanistic studies indicate that CEP-1347 inhibits c-Jun and NF-κB activity in ER^+^ breast cancer cells, and that at least some of its effects are mediated through inhibition of these pathways. Consistent with these data, overexpression of c-Jun partially rescues apoptosis induced by CEP-1347. Our data, taken together, indicate that MLK activity is required for proliferation and survival of ER^+^ breast cancer cells, and suggest that MLK inhibitors such as CEP-1347 could be potential therapeutic agents for ER^+^ breast tumors, either alone or in combination with existing therapies.

## RESULTS

### MLK inhibition reduces cell viability and induces morphological changes in breast cancer cells

**Figure 1:**
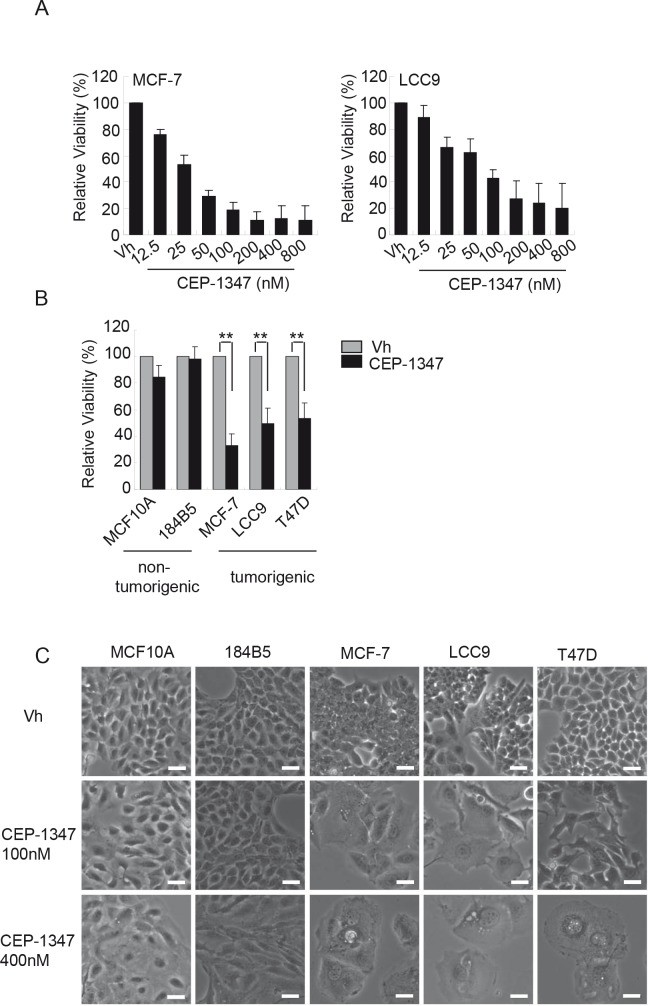
The effects of CEP-1347 on cell viability and morphology (A) Dose response curve in MCF-7 and LCC9 cells. Cells were treated with vehicle (vh) or the indicated concentration of CEP-1347 for 6 days, and viability was measured as described in Materials and Methods. Results shown are the mean +/−SD of 3 independent experiments, each done in triplicate. (B) Cells were treated with vehicle or 100 nM CEP-1347 for 6 days and assayed as described in Materials and Methods. Results shown are the mean +/−SD of 3 independent experiments, each done in triplicate. **p<0.01 (C) Cells were treated with vehicle or the indicated concentration of CEP-1347 for 6 days, and images were collected at 100X magnification. Bar = 30μm.

To determine if inhibition of MLK activity affects the viability of ER^+^ breast cancer cells, dose response assays were performed using the well-characterized, estrogen-sensitive MCF-7 breast cancer cell line and its estrogen-independent, antiestrogen-resistant derivative MCF-7/LCC9 (LCC9) [27]. Cell viability measurements after six days of treatment with various concentrations of CEP-1347 revealed that both MCF-7 and LCC-9 cells are sensitive to CEP-1347 (Figure 1A). Based on these results, 100 nM CEP-1347 was selected as the minimum effective concentration for subsequent experiments unless otherwise noted. To compare the sensitivity of breast cancer vs. non-tumorigenic mammary epithelial cells to CEP-1347, two non-tumorigenic lines, MCF10A and 184B5, and three ER^+^ breast cancer cell lines, MCF-7, LCC9 and T47D, were examined. As shown in Figure 1B, CEP-1347 treatment profoundly decreased viability of all three breast cancer cell lines, but had little effect on non-tumorigenic mammary epithelial cell lines. Imaging of CEP-1347-treated cells revealed distinct morphological changes in the breast cancer cells compared to the non-tumorigenic cells (Figure 1C). After treatment with 100 nM CEP-1347, MCF-7 and LCC9 cells became uniformly large and flat. A similar, though not as dramatic, effect was observed in T47D cells treated with 100 nM CEP-1347. At 400 nM CEP-1347, only a few large, flat cells containing multiple vacuoles remained in all tumor cell lines whereas only a minor effect was observed in the non-tumorigenic cell lines.

### MLK inhibition causes G2/M arrest in ER^+^ breast cancer cell lines

**Figure 2:**
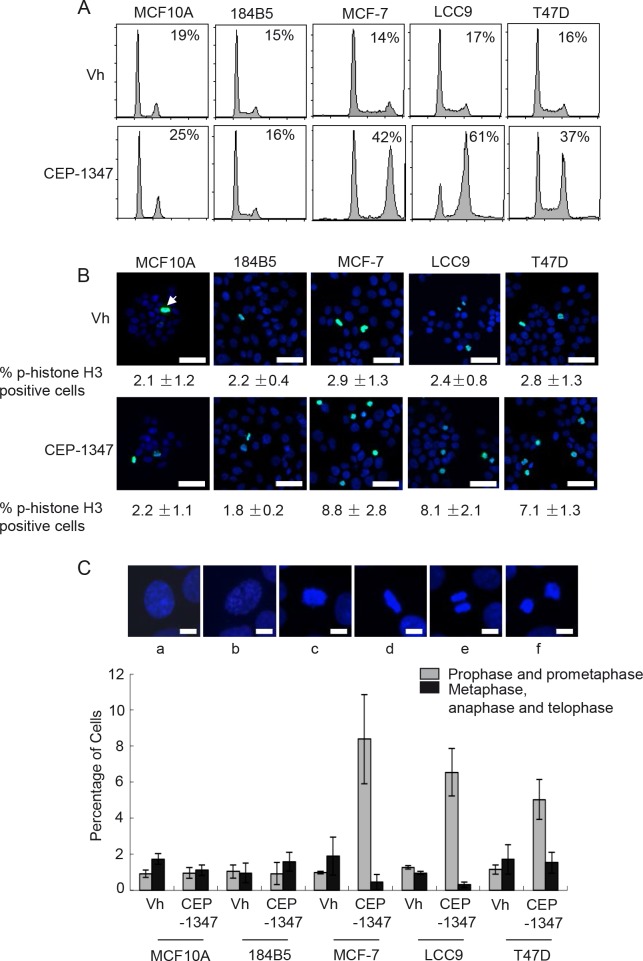
MLK inhibition causes G2/M arrest in breast cancer but not non-tumorigenic mammary epithelial cell lines Cells were treated with vehicle (vh) or CEP-1347 (100 nM) for 24 h. (A) DNA content was measured by flow cytometry as described in Materials and Methods. The percentage of cells in G2/M phases is shown. (B) Phospho-histone H3 (green) and DAPI (blue). The average percentage of phospho-histone H3 positive cells in three independent experiments is shown below each image. Bar= 30 μm (C) Cells were fixed and stained with DAPI to visualize DNA, and their cell cycle phase was identified based on the staining pattern. Representative pictures of cells in different cell cycle phases are shown. a: interphase, b: prophase, c: pro-metaphase, d: metaphase, e: anaphase, f: telophase. Bar=10μm. The percentage of mitotic cells in prophase/prometaphase, or metaphase/anaphase/telophase was determined after 24 h of CEP-1347 treatment. The results shown are the mean +/− SD of 3 independent experiments.

To examine the mechanism by which CEP-1347 decreases viability in breast cancer cells, non-tumorigenic mammary epithelial and breast cancer cell lines were treated with CEP-1347 for 24 h and analyzed for DNA content by flow cytometry. As shown in Figure 2, CEP-1347 treatment caused a large increase in the percentage of MCF-7, LCC9 and T47D cells in G2/M phase, but no significant accumulation of MCF10A or 184B5 cells in G2/M phase was detected (Figure 2A). To distinguish between G2 phase and mitosis, vehicle and CEP-1347 treated cells were stained for the mitotic marker phospho-histone H3 (Figure 2B). No change in the percentage of phospho-histone H3 positive cells was observed upon CEP-1347 treatment of the non-tumorigenic mammary epithelial cell lines. In contrast, the percentage of phospho-histone H3 positive breast cancer cells increased significantly, although it did not approach the percentage of cells in G2/M phase determined by flow cytometry. For example, the percentage of CEP-1347 treated LCC9 cells that were positive for phospho-H3 was 9%, but 61% had a G2/M DNA content. These results suggest that the primary effect of CEP-1347 is to block cells in G2, but that this arrest is not complete and some cells enter mitosis.

The distribution of cells in various mitotic stages was evaluated by staining DNA with DAPI and examining chromosome morphology. Representative images and quantification of cells in the stages of mitosis are shown in Figure 2C. CEP-1347 had no effect on the percentage of non-tumorigenic mammary epithelial cells in prophase/prometaphase or metaphase/telophase/anaphase. In contrast, the percentage of ER^+^ breast cancer cells in prophase/prometaphase was increased by CEP-1347 treatment, and the percentage in metaphase/telophase/anaphase was decreased. We therefore conclude that breast cancer cells that escape the initial CEP-1347-induced G2/M arrest are blocked prior to entering metaphase.

**Figure 3:**
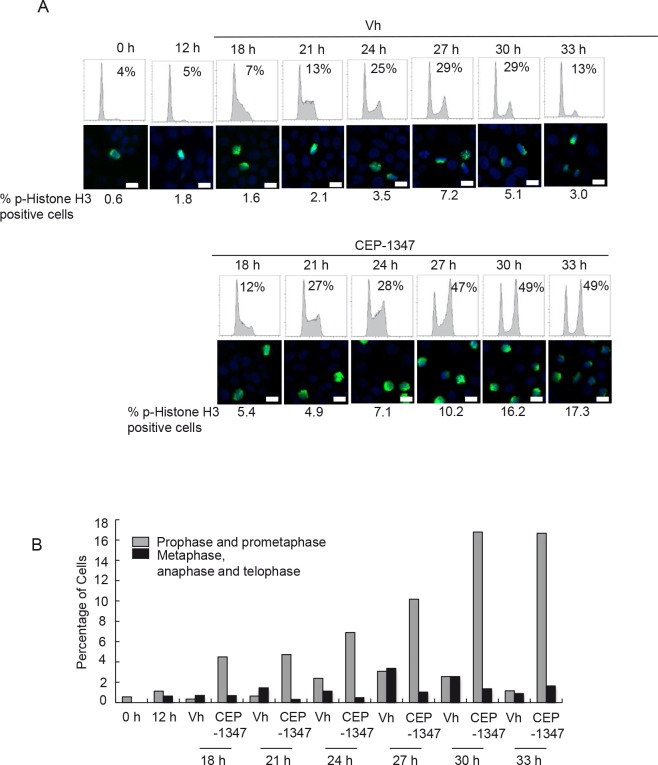
CEP-1347 treatment of synchronized MCF-7 cells causes G2 and M phase arrest MCF-7 cells were synchronized in G0/G1 phase by 48 h of ICI 182,780 (ICI) treatment, released back into the cell cycle by E2 treatment for 12 h, then incubated with or without CEP-1347 for an additional 6-21 h. (A) *Upper*: cells were collected at the indicated time points after E2 release, DNA was stained with propidium iodide, and DNA content was determined by flow cytometry. The percentage of cells in G2/M phase is indicated. *Lower*: phospho-histone H3 (green) and DAPI (blue) staining at the indicated time points. The percentage of phospho-histone H3 positive cells is shown below each image. Bar = 10 μm (B) Graph showing the percentage of prophase/prometaphase, or metaphase/anaphase/telophase cells at the indicated times after E2 release.

The effects of CEP-1347 on synchronized cells were also investigated. MCF-7 cells were pre-treated with the antiestrogen ICI 182,780 (ICI) for 48 h to induce a G0/G1 arrest, and then released into the cell cycle with E2 treatment. Twelve hours after E2 release, CEP-1347 or vehicle was added to the cultures. As shown in Figure 3, more than 90% of the cells were in G0/G1 phase after ICI treatment (0 h), and phospho-histone H3 positive cells were rarely observed at this time point. In vehicle-treated cultures, the percentage of cells in G2/M was 29% at 27 and 30 h and, by 33 h, cells had re-entered G1 with only 13% remaining in G2/M. In contrast, in CEP-1347-treated cultures, 47% of cells were in G2/M at 27 h, and this percentage did not change significantly at 30 or 33 h, indicating that cells were not completing mitosis/cytokinesis. Phospho-histone H3 staining showed a similar pattern. The percentage of vehicle-treated cells that were positive for phospho-histone H3 peaked at 7% at 27 h, then decreased at 30 and 33 h. In contrast, the percentage of phospho-histone H3 positive cells in CEP-1347-treated cultures continued to increase after 27 h, reaching 17% at 33 h. Analysis of chromosome morphology showed that metaphase cells were present at the highest levels at 27 h post E2 release in vehicle-treated cells, but were rarely observed at any time point in CEP-1347-treated cells Figure 3B. These data confirm that CEP-1347 treatment of ER^+^ breast cancer cells blocks both the G2/M and the prometaphase to metaphase transitions.

### Inhibition of MLK activity induces apoptosis in ER^+^ breast cancer cells

**Figure 4:**
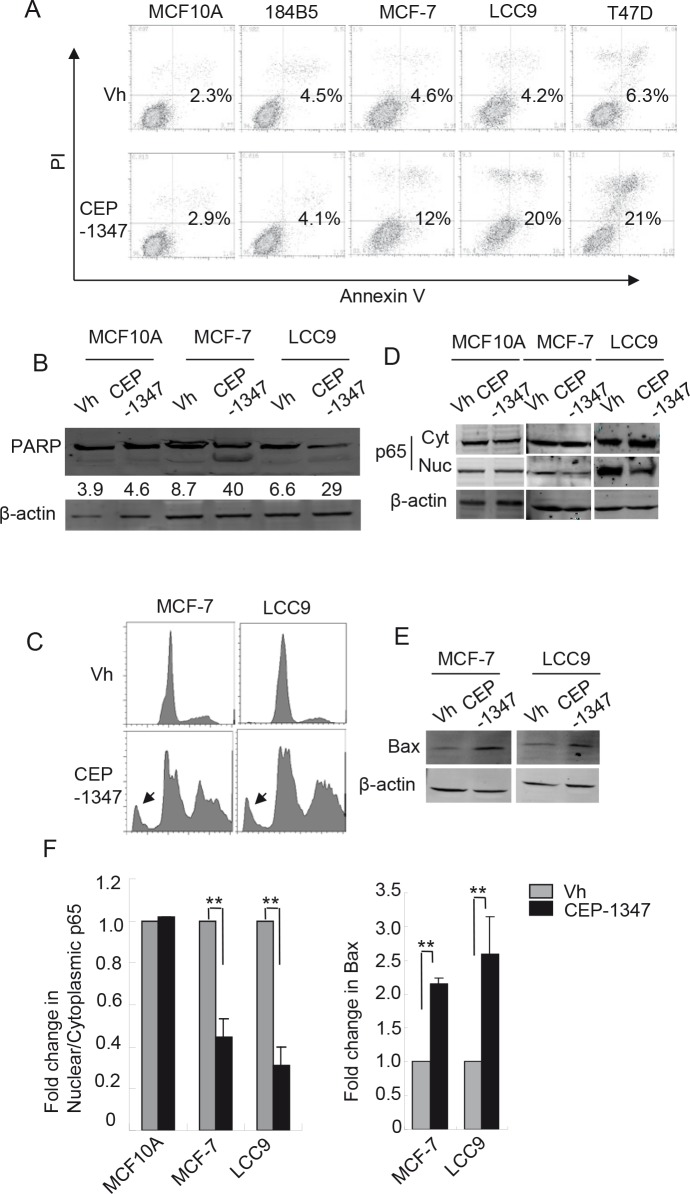
MLK inhibition induces apoptosis in breast cancer cells but not in non-tumorigenic cells Cells were treated with vehicle or 100 nM CEP-1347 for 48 h, and analyzed as follows: (A) Apoptotic cells were identified by annexin V and PI staining and measured by flow cytometry as described in Materials and Methods. Numbers shown represent the percentage of annexin V positive cells. (B) Cellular lysates were prepared and analyzed by western blotting using an antibody that recognizes both intact PARP (upper band) and cleaved PARP (lower band). The percentage of cleaved PARP is shown. Blots shown are representative of two independent experiments. (C) Flow cytometry of DNA content showing both sub-G1 (arrow) and >4n DNA content in MCF-7 and LCC9 cells treated with CEP-1347. (D) Cytoplasmic and nuclear extracts were prepared and analyzed by western blotting with anti-p65 antibody. (E) Western blot showing levels of the pro-apoptotic protein, Bax. (F) Quantitation of the fold change in the ratio of nuclear to cytoplasmic p65 and in Bax levels in response to CEP-1347 treatment relative to vehicle control. n=2 for MCF10A and n=3 for MCF-7 and LCC9. **p<0.01

To determine if CEP-1347 triggers apoptosis, cells were treated with vehicle or CEP-1347 for 48 h and stained for both annexin V binding and DNA content. As shown in Figure 4A, CEP-1347 treatment increased the percentage of apoptotic cells in all three breast cancer cell lines, but not in non-tumorigenic MCF10A or 184B5 cells. Consistent with these data, increased PARP cleavage was observed in CEP-1347-treated MCF-7 and LCC9 cells but not in MCF10A cells (Figure 4B). In addition, DNA content analysis of MCF-7 and LCC9 cells revealed a large increase in the sub-G1 fraction after CEP-1347 treatment (Figure 4C). Interestingly, a significant increase in cells with >4n DNA content was also observed, which suggests that the CEP-1347-induced G2/M arrest shown in Figure 2 is incomplete, and that some cells undergo an abnormal mitosis/cytokinesis followed by additional rounds of DNA synthesis (Figure 4C).

NF-κB and Bax, two proteins with established roles in apoptosis and cell survival, were examined in order to investigate the pathways associated with CEP-1347-induced apoptosis. The transcription factor NF-κB is involved in multiple cellular activities and is generally considered to promote pro-survival signaling. Its activity is regulated by subcellular localization, with nuclear p65 representing the active form [28]. As shown in Figure 4D and 4F, CEP-1347 treatment inhibited the nuclear localization of p65 in breast cancer cells, but not in non-tumorigenic MCF10A cells. Expression of Bax, a widely used marker of apoptosis, is negatively regulated by NF-κB. As predicted by the decrease in nuclear p65 in CEP-1347-treated breast cancer cells, Bax expression increased 2- to 3-fold (Figure 4E and 4F) under these conditions. These results are consistent with a model in which MLK inhibition results in decreased NF-κB activity, which, in turn, leads to increased Bax expression and apoptosis.

### MLK inhibition impairs the JNK pathway but has little effect on the p38 and ERK pathways in ER^+^ breast cancer cells

**Figure 5:**
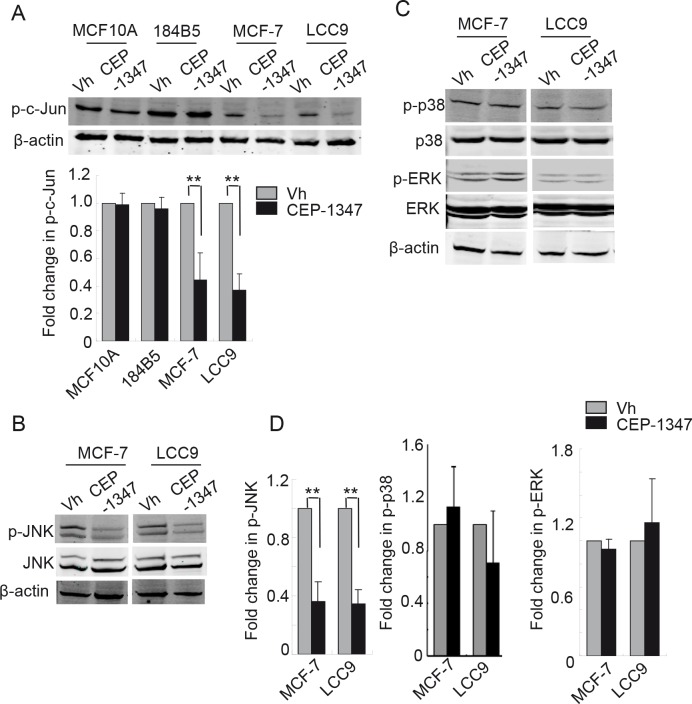
CEP-1347 treatment decreases JNK activity and c-Jun phosphorylation in MCF-7 and LCC9 cells Cells were treated with vehicle or 100 nM CEP-1347 for 48 h. Cellular lysates were prepared and analyzed by western blotting using the indicated antibodies. Actin was used as loading control. (A) Levels of phospho-c-Jun in vehicle and CEP-1347 treated cells. *Upper*: representative blots. *Lower*: quantitative analysis of p-c-Jun levels, normalized to β-actin levels. Results represent the mean +/− SD of three independent experiments. **p<0.01 (B) Total and phospho-JNK levels in MCF-7 and LCC9 cells. (C) Total and phospho-p38 and -ERK levels in MCF-7 and LCC9 cells (D) Quantitative analysis of p-JNK, p-p38 and p-ERK levels normalized to total JNK, p38 and ERK, respectively, in control and CEP-1347 treated cells. The results shown represent the mean +/− SD of 3 independent experiments. **p<0.01

Depending on the cell type and conditions, MLKs can signal via multiple MAPKs including JNKs, p38 and ERKs. To determine which MAPKs are affected by CEP-1347 treatment of ER^+^ breast cancer cells, we assessed MAPK activity by western blotting using phospho-specific antibodies. As shown in Figure 5A, CEP-1347 treatment blocked phosphorylation of the JNK substrate c-Jun in MCF-7 and LCC9 cells, but not in MCF10A or 184B5 cells. In agreement with these findings, the levels of phospho-JNK (Figure 5B, 5D) decreased more than 2-fold in CEP-1347-treated breast cancer cells. In contrast to the effect on JNK activity, CEP-1347 treatment did not affect either p38 or ERK activity (Figure 5C and 5D).

### Inhibition of JNK activity partially mimics the effects of CEP-1347

**Figure 6:**
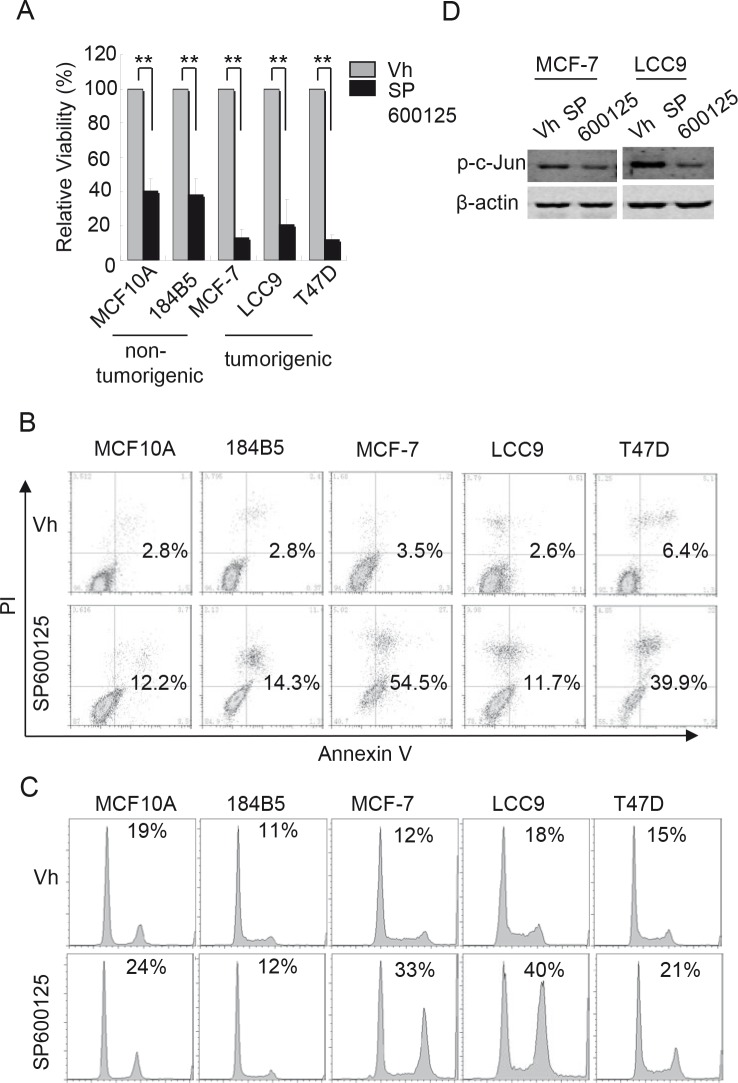
Effects of JNK inhibition on cell viability, apoptosis, and cell cycle The indicated cell lines were treated with vehicle or 15 μM SP600125 as described in Materials and Methods. (A) Cell viability after 6 days of treatment. Results shown are the mean +/−SD of 3 independent experiments. **p<0.01 (B) Cells were treated for 48 h and apoptotic cells were identified by flow cytometry after annexin V and PI staining. The percentage of annexin V positive cells is indicated. (C) Cells were treated for 24 h and DNA content was measured flow cytometry after PI staining. The percentage of cells in G2/M phase is indicated. (D) Phospho-c-Jun levels in MCF-7 and LCC9 cells after treatment with vehicle or SP600125 for 48 h.

If the effects of CEP-1347 on breast cancer cells are due to decreased JNK activity, then direct inhibition of JNK should lead to similar changes in cell cycle and survival. As shown in Figure 6A, treatment with the JNK inhibitor SP600125 decreased cell viability in both non-tumorigenic and breast cancer cell lines. Consistent with the decreased viability, SP600125 treatment induced apoptosis in both non-tumorigenic mammary epithelial cells and breast cancer cells, as determined by annexin V staining (Figure 6B). However, flow cytometric analysis showed that a 24 h SP600125 treatment caused an accumulation of breast cancer cells in G2/M, but had no such effect on non-tumorigenic cells (Figure 6C).

### Over-expression of c-Jun in breast cancer cells rescues apoptosis induced by CEP-1347

**Figure 7:**
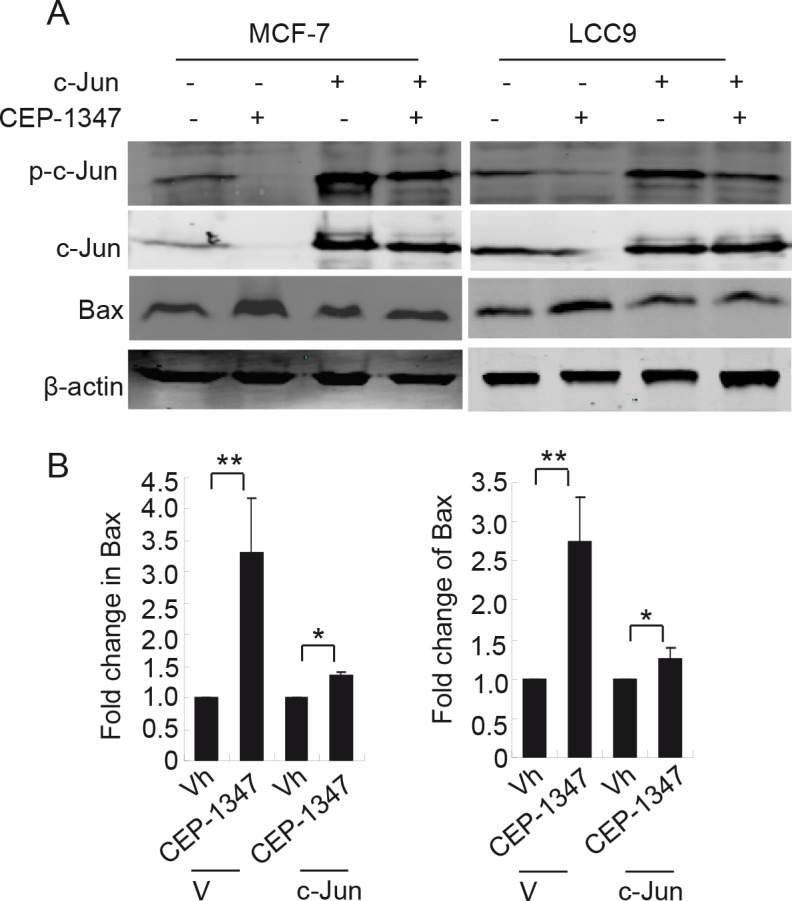
Overexpression of c-Jun suppresses CEP-1347- induced Bax expression Cells were transfected with pcDNA3.1 (V) or pcDNA 3.1/c-Jun (c-Jun) for 24 h, then treated with vehicle or CEP-1347 for an additional 2 days. (A) Cell lysates were analyzed by western blotting for levels of p-c-Jun, total c-Jun and Bax. Actin was used as loading control. (B) Fold change in Bax levels in CEP-1347-treated cultures relative to vehicle treated cultures. The results shown represent the mean +/− SD of 3 independent experiments. *p<0.05, **p<0.01.

To determine if JNK signaling through AP-1 promotes survival of ER^+^ breast cancer cells, we examined whether overexpression of c-Jun cells could rescue the CEP-1347-induced apoptosis in MCF-7 and LCC9 cells. Twenty-four hours post transfection with vector or c-Jun cDNA, cells were treated with CEP-1347 for 48 h, and Bax protein levels were examined as a measure of apoptosis. As shown in Figure 7, overexpression of c-Jun largely prevented the CEP-1347-induced increase in Bax expression, suggesting that the apoptosis of ER^+^ breast cancer cells induced by CEP-1347 is a result of decreased JNK/c-Jun signaling.

## DISCUSSION

Endocrine resistance in ER-positive breast cancer is often accompanied by upregulation of receptor tyrosine kinases or their downstream signaling pathways, and targeting such pathways has been proposed as an approach to treat or prevent endocrine resistant tumors. Since multiple cell surface receptors can contribute to resistance, an ideal target would be one that serves as an intracellular signaling hub for several different pathways. MLKs act downstream of multiple cell surface receptors and have the potential to activate multiple MAPKs as well as NF-κB. Previous experiments demonstrated that CEP-1347 suppressed proliferation of Ras-transformed NIH-3T3 cells but not normal cells [29], and that the MLK inhibitor CEP-11004 induced a G2/M arrest in HeLa cervical carcinoma cells and in Ras-transformed NIH3T3 cells but not in non-transformed cells [30]. However, the pathways affected by these inhibitors and the basis their specificity for tumorigenic cells were not examined in these studies.

In this report we investigated the effects and mode of action of the MLK inhibitor CEP-1347 in three ER^+^ breast cancer and two non-tumorigenic mammary epithelial cell lines. Treatment with CEP-1347 significantly reduced viability of all ER^+^ breast cancer cell lines, but had no such effect in non-tumorigenic mammary epithelial cell lines. The decrease in viable breast cancer cells was shown to be due to both cell cycle arrest and increased apoptosis. After 24 h of CEP-1347 treatment, there was no effect on the cell cycle of non-tumorigenic cells, but tumor cells accumulated in G2 and early M phase. The G2/M arrest induced by CEP-1347 was not absolute, since by 48 h after treatment there was an increase in cells with >4N DNA content, indicating that a population of cells had undergone additional rounds of DNA synthesis. Analysis of tumor cells treated for 48 h also revealed cells with <2N DNA content, which is suggestive of apoptosis. The increase in apoptotic tumor cells was confirmed by annexin V staining, PARP cleavage, decreased NF-κB activity, and increased Bax expression. It is interesting to note that the cell cycle and apoptotic effects of CEP-1347 do not completely depend on p53, since one of the breast cancer cell lines examined, T47D, is p53 deficient [31].

MLK3 is the best characterized member of the MLK family, but silencing of MLK3 expression using siRNA duplexes did not decrease viability of MCF-7 cells (data not shown), suggesting that MLK3 is not the sole target of CEP-1347 in ER^+^ breast cancer cells. Since reagents are not available to examine the role of each member of the MLK family, we examined the impact of CEP-1347 on ERK, JNK and p38, established downstream targets of MLKs. CEP-1347 treatment did not affect the levels of total or phosphorylated ERK or p38 in either MCF-7 or in the endocrine resistant derivative LCC9 cell line. In contrast, there was a significant decrease in JNK activity in these two tumor cell lines, but not in the non-tumorigenic cell lines, suggesting that JNK activation is independent of MLKs in non-tumorigenic mammary epithelial cells, and that other MAP3Ks are required for JNK activation in these cells.

Decreased JNK activity in ER^+^ breast cancer cells likely contributes to the cell cycle and apoptotic responses observed upon CEP-1347 treatment, since the JNK inhibitor SP600125 mimicked the cell cycle and apoptotic effects of CEP-1347, and overexpression of c-Jun prevented the CEP-1347-induced increase in Bax expression. The roles of JNK1 and JNK2 in cell proliferation and survival are complex, and the two isoforms likely function in a cell type and stimulus specific manner [32, 33]. Since our experiments did not distinguish between JNK1 and JNK2, either or both isoforms may be mediating the effects of MLKs in ER^+^ breast cancer cells. The decrease in NF-κB activity upon CEP-1347 treatment may result from the decreased JNK activity or may be an independent effect of CEP-1347, and it may also contribute to cell cycle arrest and increased apoptosis observed. This is especially interesting, since increased NF-B signaling has been implicated in antiestrogen resistance [34, 35].

In summary, our results indicate that proliferation and survival of ER-positive breast cancer cells are highly dependent upon signaling emanating from MLKs to JNK and/or NF-κB. In contrast, JNK and NF-κB activities are independent of MLKs in non-tumorigenic mammary cells, suggesting that they depend on other MAP3Ks. MLKs may therefore provide novel therapeutic targets for ER^+^ breast cancer. The breast cancer cell lines used in this study included one (T47D) with mutant p53 and one (LCC9) with acquired endocrine resistance, indicating that CEP-1347 or other MLK inhibitors may provide useful therapeutics for a wide range of ER^+^ breast tumors. MLKs have also been implicated in ER-negative breast cancer [18, 20, 21] and other tumor types [15, 19], suggesting that MLK-targeted therapies might also be useful for a wide variety of cancers. CEP-1347 was originally developed as a potential therapy for neurodegenerative diseases, since it protects neuronal cells from apoptosis triggered by neurotrophic factor withdrawal [36]. It progressed through phase II-III clinical trials for Parkinson's disease, where it lacked efficacy in delaying disease progression [37]. The serum levels achieved in the Parkinson's trials were 20-200 ng/ml, and the concentration used in our experiments is well within this range. At these concentrations, CEP-1347 displayed minimal toxicity in patients [38], supporting our finding that it is not toxic to normal cells. The fact that pharmacological doses of CEP-1347 can be achieved in people without significant adverse effects suggest that it, or other MLK inhibitors, could be rapidly developed as breast cancer therapeutics.

## METHODS

### Cell lines, cell culture and transfection

MCF10A and 184B5 cells were obtained from the ATCC. MCF-7 and MCF-7/LCC9 cells were obtained from Dr. Robert Clarke at the Lombardi Comprehensive Cancer Center at Georgetown University. T47D cells were obtained from Dr. Sandra Haslam at Michigan State University.

MCF-7, MCF7/LCC9 and 184B5 cells were maintained in improved modified Eagle's medium (Invitrogen) supplemented with 5% fetal bovine serum (FBS) (HyClone), penicillin (100 units/ml), and streptomycin (100 units/ml) (Invitrogen) and cultured at 37°C with 5% CO_2_. T47D cells were grown in DMEM/F12 (Invitrogen). MCF10A cells were maintained in medium composed of DMEM/F12 supplemented with 5% horse serum, 20 ng/ml epidermal growth factor (EGF), 10 μg/ml insulin, 0.5 μg/ml hydrocortisone (Sigma), 100 ng/ml cholera toxin (Cambrex), and 100 units/ml penicillin and 100 units/ml streptomycin.

For transfection, cells were plated in 6 cm dishes (8×10^5^ cells/dish), cultured 24 h and transfected with 4 μg of pcDNA 3.1 or pcDNA3.1-c-Jun using Lipofectamine 2000 (Invitrogen) according to the manufacturer's instructions.

### Reagents and antibodies

CEP-1347 was generously provided by Cephalon, a wholly owned subsidiary of Teva Pharmaceuticals, and SP600125 was from Calbiochem. ICI 182,780 was purchased from Tocris Bioscience Inc. 17β–estradiol (E2) was from Sigma-Aldrich. Antibodies against Bax (2772), PARP (9542), phospho-ERK (9106), phospho-JNK (9255), and phospho-p38 (9216), were from Cell Signaling Biotechnology, Inc. Antibodies to phospho-c-Jun (sc-822), c-Jun (sc-1694), ERK1/2 (sc-93), JNK1/2 (sc-475), p38 (sc-535) and NF-κB p65 (sc-372-G), were from Santa Cruz Biotechnology. Anti-beta-actin antibody (A4700) was from Sigma. The anti-phosphorylated histone H3 (Ser10) antibody was a gift from Dr. Min-Hao Kuo. The fluorescence-labeled secondary antibodies used were: IRDye 800CW donkey anti-mouse (926-32212) and IRDye 680 donkey anti-rabbit IgG (926-32223), both from Li-COR Biosciences, and Alexa Flour 680 donkey anti-goat IgG (A-21084) and Alexa Flour 488 donkey anti-rabbit IgG, both from Invitrogen.

### Cell viability assay

Cells were plated at a density of 5,000 cells/well in 96-well plates and treated with vehicle, CEP-1347 or SP600125 the following day. Fresh medium was added on day 3. On day 6, CCK-8 reagent was added to the wells and absorbance at 450 nm was measured after 2 h of incubation following the manufacturer's instructions (Dojindo Molecular Technologies). The absorbance of vehicle treated wells was defined as 100% for each cell line.

### Phase contrast microscopy

The overall appearance of vehicle- or CEP-1347- treated cells was examined by phase contrast microscopy (Nikon) on day 6 of treatment, and phase-contrast images were taken with a Photomatrix camera.

### Flow cytometry

For cell cycle analysis, cells were trypsinized, washed with phosphate-buffered saline (PBS) and fixed in 70% ethanol overnight at -20oc, and then washed two times in PBS. Cells were resuspended and incubated in PBS containing 50 μg/ml propidium iodide (PI) and 100 μg/ml RNaseA, for 15 minutes at 37oc. For apoptosis assays, cells were trypsinized, washed with PBS and then incubated with FITC annexin V and PI according to the manufacturer's instructions (BD Bioscience). Cells were analyzed using a FACS Vantage flow cytometer, and data were analyzed using FlowJo software (Tree Star). Ten thousand events were analyzed for each sample.

### Indirect immunofluorescence staining

Cells were plated and grown on glass coverslips, and treated as described. After treatment, cells were washed with PBS, fixed with 4% paraformaldehyde in PEM buffer (0.1 M Pipes, l mM EGTA, 1 mM MgCl_2_, pH 6.9) for 10 min, and permeabilized with 0.5% Triton X-100 in PEM for 10 min. Fixed, permeabilized cells were rinsed in PBS, blocked in 3% bovine serum albumin (BSA) for 30 min, then incubated with anti-phosphorylated histone H3 antibody for 1 h. After three washes with PBS, cells were incubated with Alexa Flour 488 donkey anti-rabbit IgG for 1 h, then rinsed three times with PBS containing 0.1% Triton X-100, and stained with DAPI (100 ng/ml) for 5 min. Images were acquired using a Nikon Eclipse TE2000-U fluorescence microscope.

### Western blot analysis

Cells were washed in cold PBS and lysed in CelLytic M lysis buffer (Sigma), supplemented with protease inhibitors (Complete Mini EDTA-free) and phosphatase inhibitors (PhosSTOP) from Roche. The protein concentration of the lysates was determined by the Bradford method (Bio-Rad). Proteins (30-80 μg) from cellular lysates were resolved on SDS–polyacrylamide gels, transferred to PVDF membranes, and probed with appropriate primary and secondary antibodies. Fluorescence was visualized and quantitated using a Li-COR Odyssey (Li-COR Biosciences).

For analysis of cytoplasmic and nuclear fractions, extracts were prepared as described by Dignam et al [39]. Briefly, treated cells were washed in PBS, resuspended in buffer A (10 mM HEPES at pH 7.9, 1.5 mM MgCl_2_, 10 mM KCl, 0.5 mM DTT, supplemented with protease and phosphatase inhibitors) and kept on ice for 15 min. Cells were lysed by the addition of Nonidet P-40 to 0.3%, then centrifuged at 2000 *g* for 10 min at 4°C. The resulting supernatants were collected as cytoplasmic extracts. Nuclear pellets were resuspended in buffer B (20 mM HEPES, pH 7.9, containing 1.5 mM MgCl_2_, 450 mM NaCl, 25% glycerol, 0.2 mM EDTA, 0.5 mM DTT, supplemented with protease and phosphatase inhibitors), agitated for 30 min at 4°C, and then centrifuged at 20000 *g* for 15 min. The resulting supernatants were collected as the nuclear extract.

### Statistical analysis

Results are expressed as the mean ± S.D. and experiments were performed at least three times unless otherwise noted. Statistical comparisons are based on Student's t test and a probability value of <0.05 was considered to be significant.
